# Supplementary material for: The Silencing of GhPIP5K2 and GhPIP5K22 Weakens Abiotic Stress Tolerance in Upland Cotton (Gossypium hirsutum)
Source: Int J Mol Sci. 2024 Jan 26;25(3):1511. doi: 10.3390/ijms25031511 (PMC10855785; doi:10.3390/ijms25031511)
Supplement: Supplementary file 1 [file ijms-25-01511-s001.zip › MS.pdf]

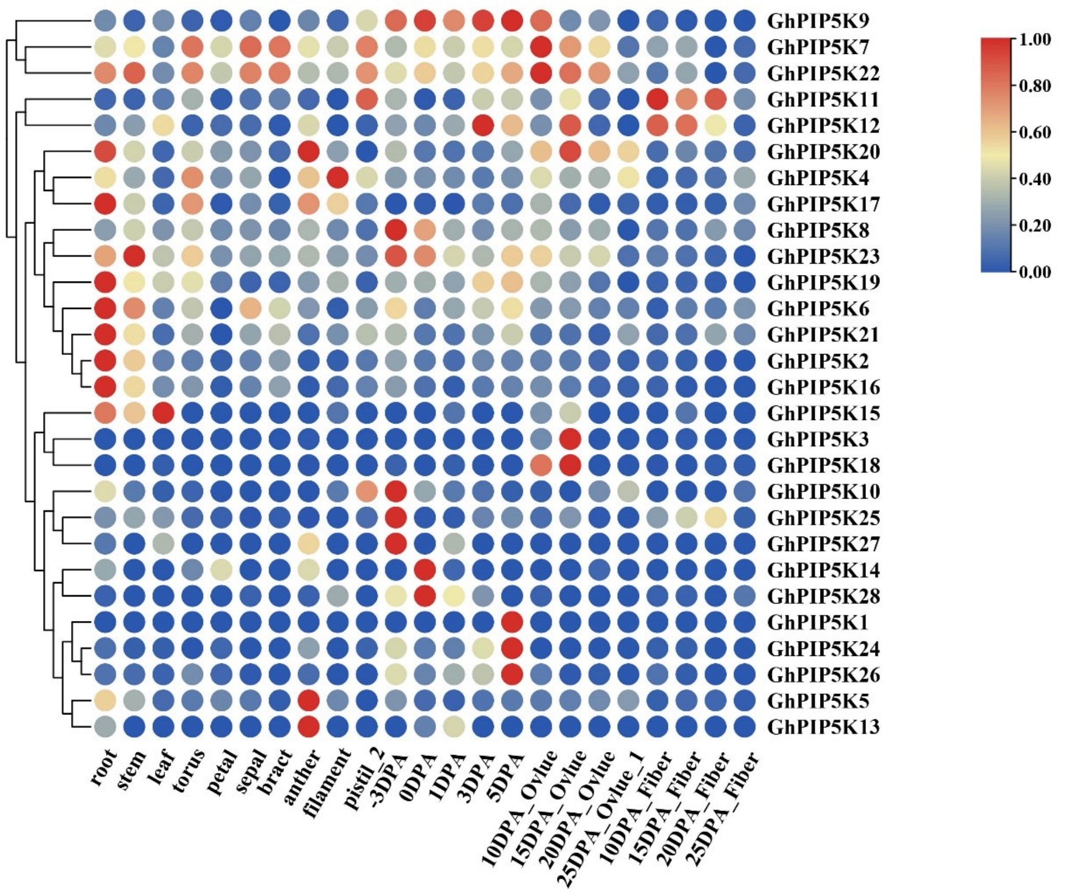

Figure S1 Transcriptome data heat maps of upland cotton *GhPIP5Ks* in different tissues.

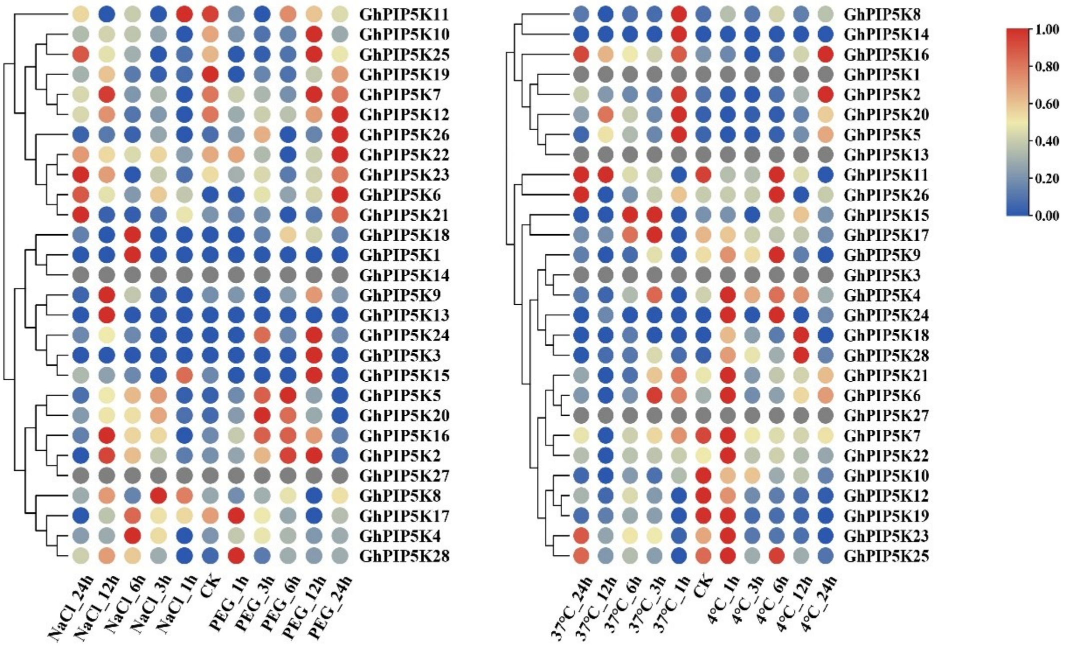

Figure S2 Transcriptome data heat map of upland cotton GhPIP5Ks under different abiotic stresses.
